# The Plastic Behavior in the Large Deflection Response of Fiber Metal Laminate Sandwich Beams under Transverse Loading

**DOI:** 10.3390/ma15020439

**Published:** 2022-01-07

**Authors:** Mingshi Wang, Jianxun Zhang, Hui Yuan, Haoyuan Guo, Wenbo Zhuang

**Affiliations:** 1 State Key Laboratory for Strength and Vibration of Mechanical Structures, School of Aerospace Engineering, Xi’an Jiaotong University, Xi’an 710049, China; mswang@xjtu.edu.cn (M.W.); hyuanxjtu@126.com (H.Y.); haoyguo2017@126.com (H.G.); zwbo1991@163.com (W.Z.); 2 State Key Laboratory of Structural Analysis for Industrial Equipment, Dalian University of Technology, Dalian 116024, China

**Keywords:** sandwich beam, fiber metal laminate, metal foam, plastic behavior, large deflection

## Abstract

The plastic behavior in the large deflection response of slender sandwich beams with fiber metal laminate (FML) face sheets and a metal foam core under transverse loading is studied. According to a modified rigid–perfectly plastic material approximation, an analytical model is developed, and simple formulae are obtained for the large deflection response of fully clamped FML sandwich beams, considering the interaction of bending and stretching. Finite element (FE) calculations are conducted, and analytical predictions capture numerical results reasonably in the plastic stage of large deflection. The influences of metal volume fraction, strength ratio of metal to composite layer, core strength, and punch size on the plastic behavior in the large deflection response of FML sandwich beams are discussed. It is suggested that, if the structural behavior of fiber-metal laminate sandwich beams is plasticity dominated, it is similar to that of metal sandwich beams. Moreover, both metal volume fraction and the strength ratio of metal to composite layer are found to be important for the plastic behavior in the large deflection response of fiber metal laminate sandwich beams, while core strength and punch size might have little influence on it.

## 1. Introduction

A sandwich structure, consisting of two face sheets with a cellular core, is a typical type of lightweight structure widely used in engineering. Several types of sandwich cores have been developed, such as honeycomb, metal foam, pyramidal truss, lattice material, and woven material [1,2,3,4,5,6,7,8,9,10,11,12,13,14,15,16,17]. Conventional face sheets are mainly made from metal or composites. Fiber metal laminates with combined metal and composite materials have shown excellent mechanical properties according to the design of layer arrangement, layer angle, and layer thickness [18,19,20]. Hence, sandwich structures with FML face sheets may perform better in carrying load and absorbing energy.

The large deflection response of sandwich beams with a foam core has been studied analytically, experimentally, and numerically. Tagarielli and Fleck [21] investigated the bending behavior of clamped sandwich beams with metal face sheets, and both the measured and the simulated large deflection response could be well predicted by the analytical model. Tagarielli et al. [22] conducted a similar study on clamped sandwich beams with composite face sheets, and good agreement was also achieved among the experimental, numerical, and theoretical results of large deflection. Considering both core strength and bending/stretch interaction, Qin and Wang [23] developed a more accurate theoretical model to predict the large deflection response for clamped sandwich beams with a metal foam core. Then, Zhang et al. [24] analytically studied the large deflection of clamped metal foam core sandwich beams, which consisted of two face sheets with two different thicknesses, considering both global deformation and local denting. Moreover, several failure modes including face sheet yield, indentation, core shear, and face sheet/core debonding have been studied for sandwich structures, and failure mechanism maps have been constructed to show the most likely failure mode [16,17,21,22].

In recent years, much attention has been paid to quasi-static and dynamic behavior of sandwich beams with FML face sheets. Dariushi and Sadighi [25] tested sandwich beams with FML face sheets and a polymer foam core subjected to three-point bending, and developed a theoretical model which could effectively predict elastic deflection. Zhang et al. [26] analytically and numerically studied the low-velocity impact response of sandwich beams with FML face sheets and metal foam core. Reyes [27] conducted low-velocity impact tests on sandwich panels with thermoplastic FML face sheets and a metal foam core, and employed an energy balance approach to predict the dynamic response. Kiratisaevee and Cantwell [28] and Liu et al. [29] tested the low-velocity impact response of metal foam core sandwich structures with glass fiber-reinforced FML face sheets, both using a drop weight tower. Liu et al. [30] carried out experimental and numerical studies on FML sandwich panels with a metal foam core subjected to high-velocity impact, and the numerical model was validated by the experimental results. Ma et al. [31] experimentally investigated the blast response of basalt FML sandwich panels with a gradient aluminum honeycomb core, and found that using basalt FML as face sheets instead of aluminum could significantly improve the blast resistance of sandwich panels. To the best of the authors’ knowledge, there is little work on large deflection of FML sandwich beams.

The objective of this study is to investigate the large deflection response of clamped slender sandwich beams with FML face sheets and a metal foam core. The problem is stated in Section 2. In Section 3, an analytical solution is obtained to predict large deflection for clamped FML sandwich beams. In Section 4, numerical calculations are carried out. In Section 5, analytical predictions are compared with numerical ones, and the influences of metal volume fraction, strength ratio of metal to composite layer, core strength, and punch size on plastic behavior are discussed. Finally, the conclusions are drawn in Section 6.

## 2. Problem Statement

**Figure 1:**
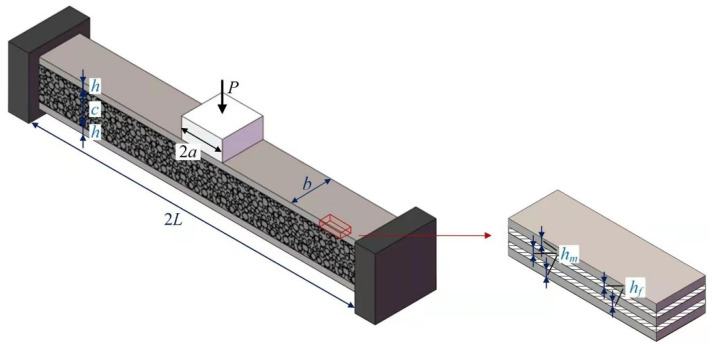
Schematic diagram of a fully clamped sandwich beam with fiber metal laminate face sheets and metal foam core transversely loaded by a flat punch at midspan.

Consider a fully clamped slender sandwich beam with FML face sheets and a metal foam core transversely loaded by a flat punch with length 2*a* and load *P* at midspan, as shown in Figure 1. The width of the rectangular sandwich cross-section is *b*, and the length of the beam is 2*L*. The FML face sheets with each thickness *h* are perfectly bonded to the metal foam core with thickness *c*. The metal foam core is assumed to be isotropic with compressive strength $\sigma_{c}$. Each FML face sheet has *n* metal layers with yield strength $\sigma_{m}$ and layer thickness $h_{m}$, separated by *n* − 1 fiber-reinforced composite layers with tensile strength $\sigma_{f}$ and layer thickness $h_{f}$. Hence, the thickness of FML face sheet is:

(1) $$h=nh_{m}+\left(n-1\right)h_{f}$$

A metal-composite layer factor is defined to describe the strength ratio of metal layer to fiber-reinforced composite layer as:

(2) $$q=\frac{\sigma_{m}}{\sigma_{f}}$$

## 3. Theoretical Analysis

Jones [32] found that, for FMLs subjected to low-velocity impact, if the structural response is dominated by plastic behavior, the FML structure will behave similar to the metal structure and, hence, the analytical solutions based on rigid–perfectly plastic assumption can be adopted to predict the dynamic response. Herein, this method is extended to study the plastic behavior in the large deflection response of sandwich beams with FML face sheets and metal foam core under quasi-static transverse loading. In the following, sandwich beams with metal face sheets are considered first.

**Figure 2:**
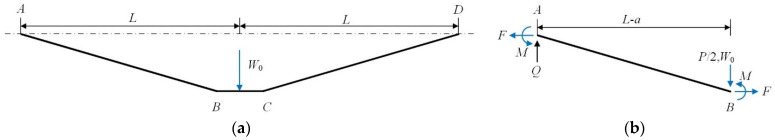
Analysis on the plastic neutral surface of a slender sandwich beam for global deformation pattern: (**a**) Transverse deflection profile; (**b**) free body diagram.

Consider the same clamped slender metal foam core sandwich beam under transverse loading, as shown in Figure 1. The only difference is that the face sheets are not made from FML but metal. For this problem, Qin and Wang [23] developed an analytical model to predict the plastic behavior of large deflection. In the analytical model, the metal of face sheets is assumed to be rigid perfectly plastic material with flow stress $\sigma_{fa}$, and the metal foam core is assumed to obey the rigid–perfectly plastic locking (*r-p-p-l*) law with yield strength (plateau stress) $\sigma_{c}$ and densification strain $\varepsilon_{D}$. Moreover, the slender sandwich beam is assumed to deform globally without local denting beneath the flat punch, and the plastic neutral surface always coincides with the geometric neutral surface due to the symmetry of the sandwich cross-sections, therefore, the transverse deflection profile shown in Figure 2a is the same as that of a slender monolithic beam under transverse loading. The equilibrium equation can be obtained from the free body diagram shown in Figure 2b,

(3) $$4M+2FW_{0}-P\left(L-a\right)=0$$

where F≈N; *N* and *M* are the plastic axial force and the plastic bending moment of the sandwich cross-sections, respectively; $W_{0}$ is the midspan deflection.

Then, to describe the relationship between *N* and *M*, Qin and Wang [23] proposed a yield criterion considering core strength effect, together with an associated plastic flow rule. Finally, combining geometric relations, Qin and Wang [23] derived the analytical solutions for the large deflection of transversely loaded metal foam core sandwich beams with metal face sheets, which are directly presented here. The relation between the normalized load $P_{}^{*}$ and the normalized midspan deflection $W_{0}^{*}$ is expressed as:

(4) $$P_{}^{*}=\frac{P}{P_{c}}=\left\{ \begin{array}{l} \frac{1}{1-\bar{a}}\left[\frac{\bar{\sigma }{\left(1+2\bar{h}\right)}^{2}}{4\bar{h}\left(1+\bar{h}\right)+\bar{\sigma }}W_{0}^{*}{}^{2}+1\right],\;\;\;\;0\leq W_{0}^{*}\leq \frac{1}{1+2\bar{h}}\\ \frac{\left(1+2\bar{h}\right)\left[\left(1+2\bar{h}\right)\left(W_{0}^{*}{}^{2}+1\right)+2\left(\bar{\sigma }-1\right)W_{0}^{*}\right]}{\left(1-\bar{a}\right)\left[4\bar{h}\left(1+\bar{h}\right)+\bar{\sigma }\right]},\;\;\;\frac{1}{1+2\bar{h}}\leq W_{0}^{*}\leq 1\\ \frac{2\left(\bar{\sigma }+2\bar{h}\right)\left(1+2\bar{h}\right)}{\left(1-\bar{a}\right)\left[4\bar{h}\left(1+\bar{h}\right)+\bar{\sigma }\right]}W_{0}^{*},\;\;\;\;\;\;\;\;W_{0}^{*}\geq 1 \end{array}\right.$$

where $P_{c}=\frac{4\sigma_{fa}bh\left(c+h\right)+\sigma_{c}bc^{2}}{L}$, $W_{0}^{*}=\frac{W_{0}}{2h+c}$, $\bar{a}=\frac{a}{L}$, $\bar{h}=\frac{h}{c}$ and $\bar{\sigma }=\frac{\sigma_{c}}{\sigma_{fa}}$. The normalized energy absorbed by the sandwich beam with metal face sheets during deformation is expressed as:

(5) $$U_{}^{*}=\left\{ \begin{array}{l} \frac{\bar{\sigma }{\left(1+2\bar{h}\right)}^{2}}{3\left(1-\bar{a}\right)\left[4\bar{h}\left(1+\bar{h}\right)+\bar{\sigma }\right]}W_{0}^{*}{}^{3}+W_{0}^{*},\;\;\;\;\;\;\;\;\;\;\;\;0\leq W_{0}^{*}\leq \frac{1}{1+2\bar{h}}\\ \frac{\left(1+2\bar{h}\right)\left\{\left(1+2\bar{h}\right)\left(W_{0}^{*}{}^{3}-S_{0}^{3}\right)+3\left(\bar{\sigma }-1\right)\left(W_{0}^{*}{}^{2}-S_{0}^{2}\right)+3\left(1+2\bar{h}\right)\left(W_{0}^{*}-S_{0}\right)\right\}}{3\left(1-\bar{a}\right)\left[4\bar{h}\left(1+\bar{h}\right)+\bar{\sigma }\right]}+S_{1},\;\frac{1}{1+2\bar{h}}\leq W_{0}^{*}\leq 1\\ \frac{\left(\bar{\sigma }+2\bar{h}\right)\left(1+2\bar{h}\right)}{\left(1-\bar{a}\right)\left[4\bar{h}\left(1+\bar{h}\right)+\bar{\sigma }\right]}\left(W_{0}^{*}{}^{2}-1\right)+S_{2},\;\;\;\;\;\;\;\;\;\;\;\;W_{0}^{*}\geq 1 \end{array}\right.$$

where $U_{}^{*}=\frac{\int_{0}^{W_{0}}P\left(W_{0}\right)dW_{0}}{P_{c}\left(2h+c\right)}$, $S_{0}=\frac{1}{1+2\bar{h}}$, $S_{1}=\frac{4\left[3\bar{h}\left(1+\bar{h}\right)+\bar{\sigma }\right]}{3\left(1-\bar{a}\right)\left(1+2\bar{h}\right)\left[4\bar{h}\left(1+\bar{h}\right)+\bar{\sigma }\right]}$ and $S_{2}=\frac{4\left[2\bar{h}{\left(1+2\bar{h}\right)}^{2}+\bar{h}\left(1+\bar{h}\right)\left(3\bar{\sigma }+1\right)+\bar{\sigma }\right]}{3\left(1-\bar{a}\right)\left(1+2\bar{h}\right)\left[4\bar{h}\left(1+\bar{h}\right)+\bar{\sigma }\right]}$.

On the basis of Equations (4) and (5), we can obtain the analytical solutions for the large deflection response of metal foam core sandwich beams with FML face sheets transversely loaded by a flat punch, by employing the method developed by Jones [32]. We assume that the present FML sandwich beams behave similar to the metal sandwich beams studied by Qin and Wang [23], and the FML could be modeled by a modified rigid–perfectly plastic material approximation with equivalent flow stress $\sigma_{fa}$. Jones [32] suggested that $\sigma_{fa}$ could be given by the following expression:

(6) $$\sigma_{fa}=f\sigma_{m}+\left(1-f\right)\sigma_{f}$$

which is called weighted strength, with metal volume fraction *f* defined as:

(7) $$f=\frac{nh_{m}}{h}\times 100\%$$

Substituting Equations (6) and (7) into Equations (4) and (5), we obtain the formulae of normalized load and normalized absorbed energy for FML sandwich beams as:

(8) $$P_{}^{*}=\left\{ \begin{array}{l} \frac{1}{1-\bar{a}\;}\left\{\frac{{\bar{\sigma }}_{c}{\left[1-f+2\left(n-1\right){\bar{h}}_{f}\right]}^{2}}{A\left(1-f\right)}W_{0}^{*}{}^{2}+1\right\},\;\;\;\;\;\;\;\;\;\;\;\;\;0\leq W_{0}^{*}\leq \frac{1-f}{1-f+2\left(n-1\right){\bar{h}}_{f}}\\ \frac{1}{\left(1-\bar{a}\right)A}\left\{\left[f\left(q-1\right)+1\right]\left[1-f+2\left(n-1\right){\bar{h}}_{f}\right]\left(W_{0}^{*}{}^{2}+1\right)\left[1+2\left(n-1\right){\bar{h}}_{f}/\left(1-f\right)\right]\right\}\\ +\frac{1}{\left(1-\bar{a}\right)A}\left\{2\left(1-f\right)\left[{\bar{\sigma }}_{c}-f\left(q-1\right)-1\right]W_{0}^{*}\left[1+2\left(n-1\right){\bar{h}}_{f}/\left(1-f\right)\right]\right\},\\ \;\frac{1-f}{1-f+2\left(n-1\right){\bar{h}}_{f}}\leq W_{0}^{*}\leq 1\\ \frac{1}{1-\bar{a}\;}\frac{2\left\{{\bar{\sigma }}_{c}\left(1-f\right)+2\left(n-1\right){\bar{h}}_{f}\left[f\left(q-1\right)+1\right]\right\}\left[1+2\left(n-1\right){\bar{h}}_{f}/\left(1-f\right)\right]}{A}W_{0}^{*},\;\;\;\;\;\;\;\;\;\;\;\;\;\;W_{0}^{*}\geq 1 \end{array}\right.$$

and

(9) $$U_{}^{*}=\left\{ \begin{array}{l} \frac{1}{1-\bar{a}}\left\{\frac{{\bar{\sigma }}_{c}{\left[1-f+2\left(n-1\right){\bar{h}}_{f}\right]}^{2}}{3A\left(1-f\right)}W_{0}^{*}{}^{3}+W_{0}^{*}\right\},\;\;\;\;\;\;\;\;\;\;\;\;\;0\leq W_{0}^{*}\leq \frac{1-f}{1-f+2\left(n-1\right){\bar{h}}_{f}}\\ \frac{1}{A\left(1-\bar{a}\right)}\left\{\left[f\left(q-1\right)+1\right]\left[1-f+2\left(n-1\right){\bar{h}}_{f}\right]\left[1+2\left(n-1\right){\bar{h}}_{f}/\left(1-f\right)\right]\left(W_{0}^{*}{}^{3}/3+W_{0}^{*}\right)\right\}\\ +\frac{1}{A\left(1-\bar{a}\right)}\left\{\left(1-f\right)\left[{\bar{\sigma }}_{c}-f\left(q-1\right)-1\right]\left[1+2\left(n-1\right){\bar{h}}_{f}/\left(1-f\right)\right]W_{0}^{*}{}^{2}+S_{3}\right\},\\ \;\frac{1-f}{1-f+2\left(n-1\right){\bar{h}}_{f}}\leq W_{0}^{*}\leq 1\\ \frac{\left\{{\bar{\sigma }}_{c}\left(1-f\right)+2\left(n-1\right){\bar{h}}_{f}\left[f\left(q-1\right)+1\right]\right\}\left[1+2\left(n-1\right){\bar{h}}_{f}/\left(1-f\right)\right]}{A\left(1-\bar{a}\right)}W_{0}^{*}{}^{2}+S_{4},\;\;\;\;\;\;\;\;\;\;\;\;\;\;W_{0}^{*}\geq 1 \end{array}\right.$$

respectively, where ${\bar{h}}_{f}=\frac{h_{f}}{c}$, ${\bar{\sigma }}_{c}=\frac{\sigma_{c}}{\sigma_{f}}$, $A=4\left(n-1\right){\bar{h}}_{f}\left[1+\frac{\left(n-1\right){\bar{h}}_{f}}{1-f}\right]\left[f\left(q-1\right)+1\right]+{\bar{\sigma }}_{c}\left(1-f\right)$, $\begin{array}{l} S_{3}=\frac{1-f}{1-f+2\left(n-1\right){\bar{h}}_{f}}\left[\frac{{\bar{\sigma }}_{c}\left(1-f\right)}{3}+A\right]\\ -\left[\frac{2\left(n-1\right){\bar{h}}_{f}}{1-f}+1\right]\left(1-f\right)\left\{\frac{{\bar{\sigma }}_{c}-2\left[f\left(q-1\right)+1\right]/3}{{\left[1-f+2\left(n-1\right){\bar{h}}_{f}\right]}^{2}}{\left(1-f\right)}^{2}+f\left(q-1\right)+1\right\} \end{array}$ and $S_{4}=\frac{{\left[1-f+2\left(n-1\right){\bar{h}}_{f}\right]}^{2}\left[f\left(q-1\right)+1\right]}{3A\left(1-f\right)\left(1-\bar{a}\right)}+\frac{S_{3}}{\left(1-\bar{a}\right)A}$.

## 4. Finite Element Analysis

FE calculations are conducted to study the large deflection of sandwich beams with FML face sheets and a metal foam core by using ABAQUS/Standard. The four-node bilinear quadrilateral plane strain elements with reduced integration (CPE4R) are selected for both the face sheets and the metal foam core. There are two in-plane translational degrees of freedom for each node, one along the length direction of the sandwich beam and another perpendicular to it. The punch is modeled as a rigid roller (*a*→0) with predefined displacement. At the two clamped ends, all displacements of nodes and the rotation degree of freedom are zero. Frictionless contact is applied between the top face sheet and the roller. “Tie” constraint is applied between the layers of the FML face sheet, and also between each face sheet and the core.

**Figure 3:**
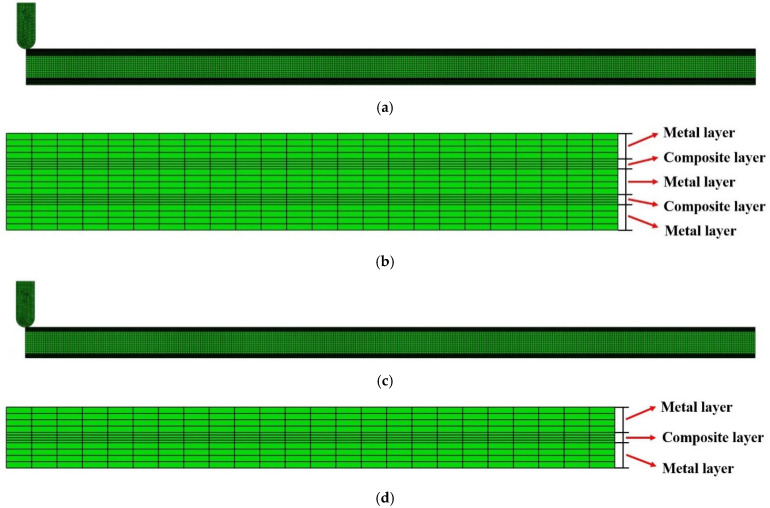
FE models of the two cases: (**a**) Half of the FML sandwich beam loaded by the roller (Case 1); (**b**) details of the FML face sheet (Case 1); (**c**) half of the FML sandwich beam loaded by the roller (Case 2); (**d**) details of the FML face sheet (Case 2).

The half span of the sandwich beam is L=200 mm and the core thickness is c=6 mm, therefore, $\bar{c}=c/L=0.03$. The thickness of each metal layer is $h_{m}=0.5\;\mathrm{mm}$, and the thickness for the fiber-reinforced composite layer is $h_{f}=0.2\;\mathrm{mm}$. The radius of the loading roller is R=2.5 mm. Two cases are calculated, different in the FML face sheets: (Case 1) *n* = 3, i.e., each FML face sheet has three metal layers and two composite layers, with a total thickness h=1.9 mm and (Case 2) *n* = 2, i.e., each FML face sheet has two metal layers and one composite layer, with a total thickness h=1.2 mm. The FE models of the two cases are shown in Figure 3.

There are 800 elements in length direction, 12 elements in height direction for the metal foam core, and 4 elements in height direction for each layer of metal or composite of the FML face sheets. Hence, the total number of elements is 41,600 for the sandwich beam of Case 1, and it is 28,800 for that of Case 2. A mesh sensitivity check reveals that additional mesh does not change the results appreciably.

**Figure 4:**
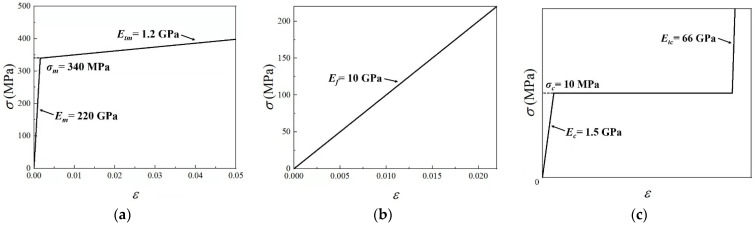
Material stress–strain curves used in FE calculations: (**a**) Metal layer of FML face sheets; (**b**) fiber-reinforced composite layer of FML face sheets; (**c**) metal foam core.

The metal layers in simulations are modeled as elastic-plastic material with linear hardening, having yield strength $\sigma_{m}=340\mathrm{MPa}$, Young’s modulus $E_{m}=220\mathrm{GPa}$, elastic Poisson’s ratio $\nu_{em}=0.3$, and tangent hardening modulus $E_{tm}=1.2\mathrm{GPa}$, as shown in Figure 4a. The material parameters of the composite layers follow the experimental data of a kind of woven glass fiber composite, which contains a quasi-isotropic fiber fabric (equal amounts of fibers in 0°, +45°, 90° and –45° directions) infused with a vinylester resin [22]. Thus, the fiber-reinforced composite layers have tensile strength $\sigma_{f}=220\mathrm{MPa}$, Young’s modulus $E_{f}=10\mathrm{GPa}$, and elastic Poisson’s ratio $\nu_{ef}=0.3$, as shown in Figure 4b.

The metal foam core is modeled by the Deshpande–Fleck constitutive model [33] implemented in ABAQUS, which allows the shape change of yield surface due to differential hardening along the hydrostatic and deviatoric axes. The yield function of the foam core is:

(10) $$\varphi =\hat{\sigma }-\sigma_{c}=0$$

where ${\hat{\sigma }}^{2}\equiv \frac{1}{1+{\left(\alpha /3\right)}^{2}}\left(\sigma_{e}^{2}+\alpha^{2}\sigma_{s}^{2}\right)$ with von Mises effective stress $\sigma_{e}\equiv \sqrt{3s_{ij}s_{ij}/2}$, the deviatoric stress $s_{ij}$, the mean stress $\sigma_{s}\equiv \sigma_{kk}/3$, and the shape factor α of the yield surface. The associated plastic flow rule is adopted and the plastic Poisson’s ratio $\nu_{p}$ is calculated by:

(11) $$\nu_{p}=-\frac{{\dot{\varepsilon }}_{{}_{22}}^{p}}{{\dot{\varepsilon }}_{{}_{11}}^{p}}=\frac{1/2-{\left(\alpha /3\right)}^{2}}{1+{\left(\alpha /3\right)}^{2}}$$

The isotropic metal foam core has yield strength (plateau stress) $\sigma_{c}=10\mathrm{MPa}$, Young’s modulus $E_{c}=1.5\;\mathrm{GPa}$, elastic Poisson’s ratio $\nu_{ec}=0.3$, plastic Poisson’s ratio $\nu_{p}=0$, and densification strain $\varepsilon_{D}=0.5$. After densification, the metal foam core is assumed to obey the linear hardening law with tangent hardening modulus $E_{tc}=66\mathrm{GPa}$, as shown in Figure 4c.

## 5. Comparison between Analytical Predictions and Numerical Results

**Figure 5:**
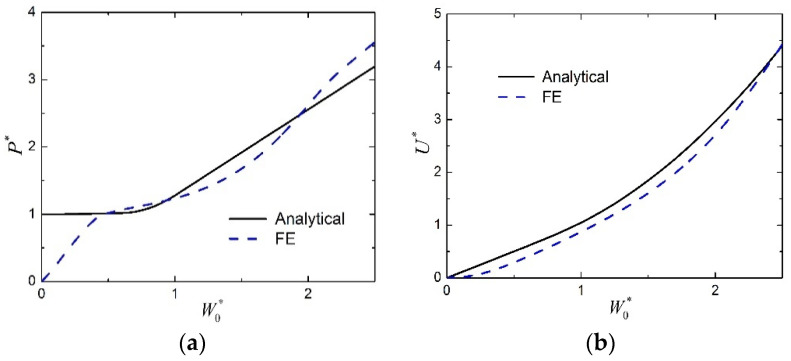
Comparison between analytical predictions and numerical results for the large deflection response of FML sandwich beams under transverse loading (Case 1): (**a**) Load versus midspan deflection; (**b**) absorbed energy versus midspan deflection.

**Figure 6:**
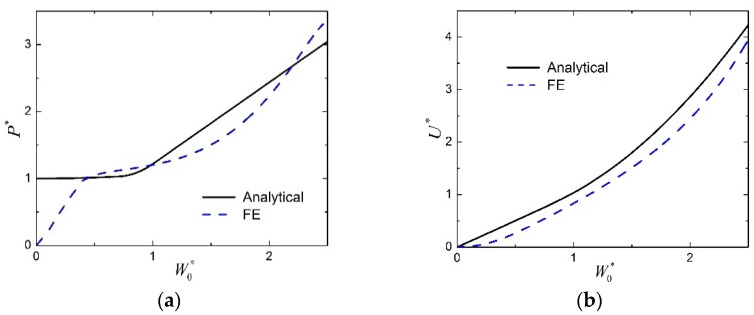
Comparison between analytical predictions and numerical results for the large deflection response of FML sandwich beams under transverse loading (Case 2): (**a**) Load versus midspan deflection; (**b**) absorbed energy versus midspan deflection.

The normalized load $P_{}^{*}$ is plotted as a function of the normalized midspan deflection $W_{0}^{*}$ for the transversely loaded FML sandwich beam of Case 1 in Figure 5a, in which both the theoretical predictions and the FE results are shown. Similar results are presented in Figure 6a for that of Case 2. It can be seen whether from Figure 5a or Figure 6a that the present analytical model cannot capture the initial elastic response due to the rigid–perfectly plastic assumption, but in the subsequent plastic stage of the large deflection response, the theoretical predictions match reasonably with the FE results. The discrepancy between the analytical predictions and the numerical results might be due to several factors which are not considered in the analytical model, including shear force and material strain hardening. In the FE calculations, these factors might have a certain range of effects on the response, and these effects are coupled and complex. The numerical results are caused by the comprehensive effects of all factors, therefore, they are sometimes higher, while sometimes lower, than the analytical predictions for the response in the plastic stage of large deflection. The relation between the normalized absorbed energy $U_{}^{*}$ and the normalized midspan deflection $W_{0}^{*}$ is shown in Figure 5b for the FML sandwich beam of Case 1, and shown in Figure 6b for that of Case 2. Reasonable agreement is also found between the analytical solutions and the numerical results during the plastic stage of the large deflection response, whether from Figure 5b or Figure 6b. Therefore, the present analytical solutions are employed to investigate the effects of several factors on the plastic behavior in the large deflection response of sandwich beams with FML face sheets and metal foam core.

## 6. Parametric Study

**Figure 7:**
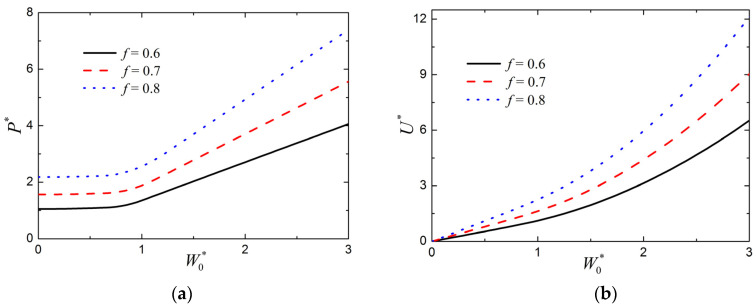
Influence of metal volume fraction *f* on the large deflection response of FML sandwich beams transversely loaded by a flat punch ($\bar{c}=0.03$, $\bar{a}=0.05$, ${\bar{h}}_{f}=0.05$, ${\bar{\sigma }}_{c}=0.1$, n=3, q=2): (**a**) Load versus midspan deflection; (**b**) absorbed energy versus midspan deflection.

For different values of metal volume fraction *f*, the relation between the normalized load $P_{}^{*}$ and the normalized midspan deflection $W_{0}^{*}$ is presented in Figure 7a; the relation between the normalized absorbed energy $U_{}^{*}$ and the normalized midspan deflection $W_{0}^{*}$ is shown in Figure 7b. Other parameters are given as $\bar{c}=0.03$, $\bar{a}=0.05$, ${\bar{h}}_{f}=0.05$, ${\bar{\sigma }}_{c}=0.1$, n=3, and q=2. It can be seen from Figure 7 that the change of metal volume fraction *f* causes significant changes both in the load–deflection relation and in the absorbed energy–deflection relation. Therefore, the metal volume fraction plays an important role in the plastic behavior of the large deflection response of FML sandwich beams.

**Figure 8:**
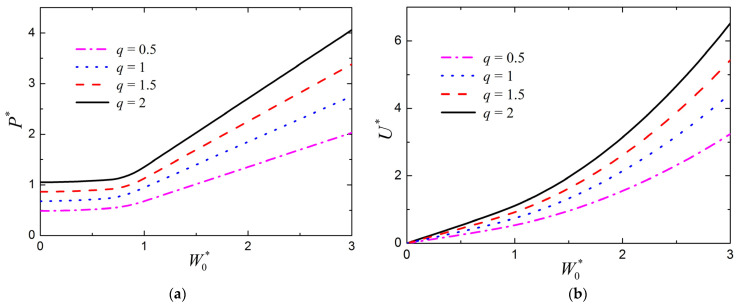
Influence of strength ratio of metal to composite layer *q* on the large deflection response of FML sandwich beams transversely loaded by a flat punch ($\bar{c}=0.03$, $\bar{a}=0.05$, ${\bar{h}}_{f}=0.05$, ${\bar{\sigma }}_{c}=0.1$, n=3, f=0.6): (**a**) Load versus midspan deflection; (**b**) absorbed energy versus midspan deflection.

The influence of the strength ratio of metal to composite layer *q* on the large deflection response of FML sandwich beams under transverse loading is shown in Figure 8a,b for the normalized load $P_{}^{*}$ versus the normalized midspan deflection $W_{0}^{*}$ and the normalized absorbed energy $U_{}^{*}$ versus the normalized midspan deflection $W_{0}^{*}$, respectively, in which $\bar{c}=0.03$, $\bar{a}=0.05$, ${\bar{h}}_{f}=0.05$, ${\bar{\sigma }}_{c}=0.1$, n=3, and f=0.6. For different values of *q*, obvious variations can be found in Figure 8, for both the load–deflection relation and the absorbed energy–deflection relation. Hence, the strength ratio of metal layer to composite layer also plays an important role in the plastic behavior of the large deflection response of FML sandwich beams.

**Figure 9:**
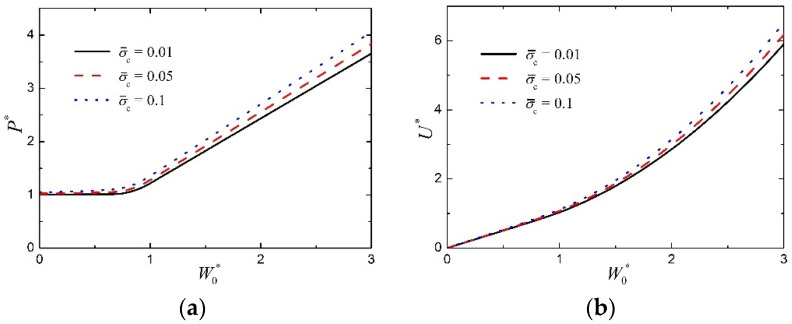
Influence of core strength on the large deflection response of FML sandwich beams transversely loaded by a flat punch ($\bar{c}=0.03$, $\bar{a}=0.05$, ${\bar{h}}_{f}=0.05$, n=3, q=2, f=0.6): (**a**) Load versus midspan deflection; (**b**) absorbed energy versus midspan deflection.

Figure 9a,b show the effect of core strength on the normalized load $P_{}^{*}$ and the normalized absorbed energy $U_{}^{*}$, as functions of the normalized midspan deflection $W_{0}^{*}$, of transversely loaded FML sandwich beams, respectively. Other parameters are given as $\bar{c}=0.03$, $\bar{a}=0.05$, ${\bar{h}}_{f}=0.05$, n=3, q=2, and f=0.6. It can be found from Figure 9 that, when core strength ${\bar{\sigma }}_{c}$ increases within a certain range, both the load-carrying and energy-absorption abilities increase a little. It seems that the effect of core strength on the plastic behavior of the large deflection response of FML sandwich beams is not significant.

**Figure 10:**
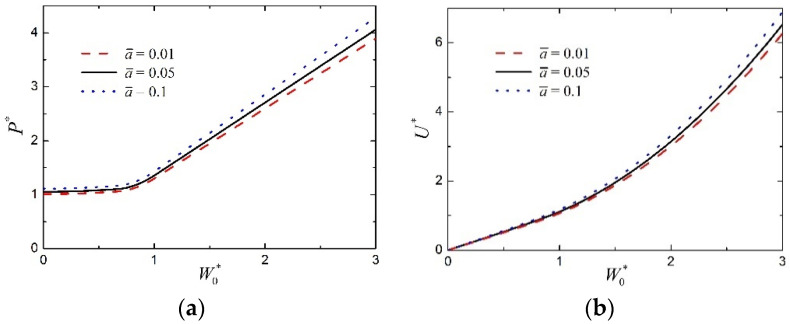
Influence of punch size on the large deflection response of FML sandwich beams transversely loaded by a flat punch ($\bar{c}=0.03$, ${\bar{h}}_{f}=0.05$, ${\bar{\sigma }}_{c}=0.1$, n=3, q=2, f=0.6): (**a**) Load versus midspan deflection; (**b**) absorbed energy versus midspan deflection.

For FML sandwich beams transversely loaded by a flat punch with different sizes, the relations of load–deflection and absorbed energy–deflection are presented in Figure 10a,b respectively, in which $\bar{c}=0.03$, ${\bar{h}}_{f}=0.05$, ${\bar{\sigma }}_{c}=0.1$, n=3, q=2, and f=0.6. From Figure 10, for a given midspan deflection, both the load and the absorbed energy increase a little with an increase in punch size $\bar{a}$. Hence, punch size has little effect on the plastic behavior of the large deflection response of FML sandwich beams.

From Equations (8) and (9), the effect of punch size $\bar{a}$ on the relations of load–deflection and absorbed energy–deflection is easy to find, i.e., both the normalized load and the normalized absorbed energy are positively proportional to $1/\left(1-\bar{a}\right)$. The beam is always much longer than the flat punch, i.e., $\bar{a}$ is always far less than one, therefore, when $\bar{a}$ increases, both the load and the absorbed energy increase a little for a given midspan deflection. However, it is hard to find the effects of metal volume fraction *f*, strength ratio of metal to composite layer *q,* and core strength ${\bar{\sigma }}_{c}$ on the relations of load–deflection and absorbed energy–deflection from Equations (8) and (9) directly. Actually, for a sandwich beam deforming globally without other modes, the two face sheets make a dominate contribution to the structural load-carrying and energy-absorption abilities; however, if other deformation modes occur, such as local denting of the core, it may not be available [24]. For the plastic behavior of the large deflection response of FML sandwich beams, only global deformation is considered in this study. Therefore, when a change of *f* or *q* leads to a change in the material properties of the FML face sheets, the plastic behavior of the large deflection response of FML sandwich beams is greatly affected, while the change of core strength has little influence.

## 7. Conclusions

The plastic behavior of the large deflection response of fully clamped slender sandwich beams with fiber metal laminate face sheets and metal foam core transversely loaded by a flat punch is investigated theoretically and numerically in this study. According to a modified rigid–perfectly plastic material approximation for the fiber metal laminate face sheets, simple formulae are given for the plastic behavior of the large deflection response of fiber metal laminate sandwich beams. Reasonable agreement is achieved between the analytical predictions and the numerical results in the plastic stage of the large deflection response, for the relations of both load–deflection and absorbed energy–deflection. It is suggested that if the structural behavior of fiber-metal laminate sandwich beams is plasticity dominated it is similar to that of metal sandwich beams. Moreover, both the metal volume fraction and the strength ratio of metal to composite layer are found to be important for the plastic behavior of the large deflection response of fiber-metal laminate sandwich beams, while core strength and punch size might have little influence on it.

Several factors including material elasticity, shear force, and material strain hardening are neglected in the present analytical model, which cause the difference between the analytical predictions and the numerical results. Moreover, other factors such as the randomness of metal foam cells, the adhesion between the layers of the FML face sheet, the adhesion between each face sheet and the core, and material failure, are not considered in the analytical model or the FE calculations. To overcome these limitations, experimental studies are suggested in the future.

## Data Availability

The data presented in this study are available on reasonable request from the corresponding author.
